# Identifying a Common Autoimmune Gene Core as a Tool for Verifying Biological Significance and Applicability of Polygenic Risk Scores

**DOI:** 10.3390/ijms27010543

**Published:** 2026-01-05

**Authors:** Victoria Sergeevna Shchekina, Nikita Aleksandrovich Batashkov, Anna Arkadievna Maznina, Julia Aleksandrovna Krupinova, Viktor Pavlovich Bogdanov, Anna Vasilievna Korobeinikova, Dmitry Igorevich Tychinin, Olga Valentinovna Glushkova, Ekaterina Sergeevna Petriaikina, Dmitry Vladimirovich Svetlichnyy, Mary Woroncow, Vladimir Sergeevich Yudin, Anton Arturovich Keskinov, Sergey Mikhailovich Yudin, Veronika Igorevna Skvortsova, Dmitry Vyacheslavovich Tabakov, Andrei Andreevich Deviatkin, Pavel Yu. Volchkov

**Affiliations:** 1 Federal State Budgetary Scientific Institution “Federal Research Center for Innovator and Emerging Biomedical and Pharmaceutical Technologies”, Moscow 125315, Russia; 2 Moscow Center for Advanced Studies, Kulakova Street 20, Moscow 123592, Russia; 3 Moscow Clinical Scientific Center N.A. A.S. Loginov, Novogireevskaya Street, 1, 1, Moscow 111123, Russia; 4 Federal State Budgetary Institution «Centre for Strategic Planning and Management of Biomedical Health Risks» of the Federal Medical and Biological Agency (Centre for Strategic Planning, of the Federal Medical and Biological Agency), Moscow 119121, Russia; 5 The Federal Medical Biological Agency (FMBA of Russia), Moscow 123182, Russia

**Keywords:** autoimmune disease, polygenic risk score, PRS, SNP

## Abstract

Polygenic autoimmune diseases (ADs) have several common features that are caused by a complex interplay of genetic and environmental factors. Common pathophysiological mechanisms include dysregulation of the immune system, chronic inflammation, and epigenetic changes influenced by external factors. For the prediction of the genetic predisposition of AD manifestation, polygenic risk scale (PRS), or polygenic scores (PGSs), are used. Use of PRSs faces several challenges such as applicability on a specific population, performance comparison, and estimation of biological relevance based on SNP number. We compared PRS with different numbers of SNPs and tried to find the common genetic core of ADs. Our analysis revealed a list of the most common altered genes, which we annotated and interpreted. Clustering of PRS based on used genes showed that clusters of ADs remained consistent across all chosen PRS sizes. We concluded that PRS size does not have an impact on biological relevance.

## 1. Introduction

Autoimmune diseases (ADs) are conditions resulting from an abnormal response of the adaptive immune system in which it mistakenly targets healthy, functional tissues of the organism as if they were foreign [1]. Over 100 types of autoimmune diseases have been identified [2]. Monogenic autoimmune diseases, such as Autoimmune polyendocrine syndrome type 1 and IPEX syndrome, result from pathogenic mutations in single genes (AIRE and FOXP3, respectively) that disrupt the function of the corresponding proteins [3]. Polygenic autoimmune diseases have several common features that are caused by a complex interplay of genetic and environmental factors [4]. These diseases arise from variations in multiple genes, often involving immune-related genes, such as the HLA region, and their onset can be influenced by multiple factors, such as gender (can be triggered during pregnancy or due to a disbalance of sex hormones) [2] and environmental triggers (infections, toxins, stress, and diet). Infections often act as triggers for autoinflammatory processes in genetically susceptible individuals through various mechanisms, such as activation of innate immune responses, molecular mimicry, and T-cell activation [5]. Despite these associations, the exact mechanisms of polygenic AD development are still unclear. Common pathophysiological mechanisms include dysregulation of the immune system, chronic inflammation, and epigenetic changes influenced by external factors. Clinically, these diseases often present with overlapping symptoms such as fatigue, joint pain, or organ-specific inflammation and progress in episodes, typically being challenging to diagnose and treat [6].

An overlap of two or more autoimmune diseases commonly manifests in a single patient [7]. Type 1 diabetes (T1D) is commonly associated with autoimmune thyroid diseases, such as Addison’s disease, celiac disease, Hashimoto’s thyroiditis, and Graves’ disease. Other ADs may also be associated with each other, particularly Sjögren’s syndrome with systemic lupus erythematosus and systemic sclerosis [8]. These conditions co-occur in 17–30% of cases. Other associated conditions include celiac disease (8%) and autoimmune gastritis (5–10%). However, conditions such as rheumatoid arthritis, Addison’s disease, and systemic lupus erythematosus are rarer, occurring in only 1.2%, 0.2%, and 1.15% of T1DM cases, respectively [9]. It was shown by Gokuladhas S. et al. that among 2065 SNPs associated with ADs, 186 were associated with two or more ADs simultaneously [10].

The Genome-Wide Association Study (GWAS) approach is commonly used to understand the genetic landscape that contributes to the development of autoimmune diseases [11]. Among tissue-specific autoimmune diseases, this type of analysis has been performed in type 1 diabetes mellitus (T1DM) and multiple sclerosis [12,13]. Genetic predisposition to autoimmune diseases can significantly increase the likelihood of developing these conditions. Different variants and their combinations can carry varying levels of risk, from low to high. Among the variants, there are predisposing alleles as well as protective alleles. For instance, the relative risk associated with individual genetic variants can be high, increasing the probability of developing diseases such as T1D several-fold [14,15,16]. Due to the frequent co-occurrence of ADs, it is plausible that they share common genomic variant signatures.

To find the biological relevance of GWAS findings, some studies clusterized ADs, based on GWAS summary statistics. This approach identified pleiotropic gene mutations that are involved in the pathogenesis of multiple diseases. In research by Demela et al. [17], GWAS results for nine autoimmune diseases were summarized and three tissue-specific clusters were identified based on their associated polymorphisms. The first cluster included gastrointestinal diseases (Crohn’s disease, ulcerative colitis, and primary sclerosing cholangitis), the second—systemic lupus erythematosus, T1D, and rheumatoid arthritis, and the third—allergic diseases. The study showed that in these clusters, changes occur at different nodes in the same immune pathways, and different immune cell patterns are involved in their pathogenesis.

For the prediction of genetic predisposition of AD manifestation, the polygenic risk scale (PRS) or polygenic score (PGS) are used [17,18,19]. PRSs are statistical models that summarize the effects of multiple genetic variants (SNPs) to predict disease risk and developed based on identified associations in GWAS studies. However, their interpretation and relevance between different samples remain a matter of debate.

The quality of the PRS depends on several factors, including:

**Sample size and diversity:** PRS that contain large and genetically diverse samples generally have better predictive power.

**Population:** PRS designed for one population may not work well for other populations due to genetic differences [20].

Additionally, PRSs face several challenges [21,22]:

**The limited power of GWAS**: GWAS studies underlying PRS often have limited statistical power, especially for rare variants or variants with small effect. This leads to many true associations of genes with the disease going unnoticed, which reduces the accuracy of PRS.

**Pleiotropy**: Many genes are involved in several biological processes. Therefore, the same genetic variant can be associated with several diseases, which makes it difficult to interpret the GWAS results and construct accurate PRS.

**Relationship without causation**: GWAS identifies statistical links between genetic variants and a disease, but does not necessarily establish a causal relationship. The association may be due to a link with the true causal variant located nearby on the chromosome.

In the literature, PRSs for various traits range from several hundred to tens of thousands of SNPs [23]. It may be assumed that a PRS based on a relatively small number of SNPs would include only highly significant variants and therefore exhibit strong predictive performance, whereas a PRS incorporating a very large number of SNPs may be less predictive. However, the number of SNPs included in a PRS is not the most important factor in its predictive quality. The most reliable way to evaluate a PRS is by assessing its predictive power on independent samples (validation). However, the comparison of even two PRSs can be challenging, as there is no universally accepted standard for the measurement of PRS effect sizes. Namely, in the PGS catalog, performance estimation can be based on the odds ratio, beta correlation, incremental AUROC, or various other factors (https://www.pgscatalog.org/). This lack of standardization complicates the interpretation and transferability of PRSs, particularly in autoimmune diseases.

To address this challenge, we selected the most common diseases for our research: rheumatoid arthritis (RA), psoriasis, Hashimoto’s thyroiditis (HT), colitis, type 1 diabetes mellitus (T1D), asthma, systemic lupus erythematosus (SLE), celiac disease, Crohn’s disease, and multiple sclerosis (Table 1). Our selection process was meticulous, aiming to encompass a range of autoimmune diseases, characterized by a generalized course, and included diseases representative of certain target affected organs or systems of organs. Additionally, we included diseases known to have an association with type 1 diabetes, thereby ensuring a comprehensive representation of autoimmune conditions with potential interconnectedness. This approach allowed us to focus our research efforts on a manageable, yet diverse set of autoimmune diseases, facilitating a more in-depth analysis of their characteristics and potential correlations.

This study aimed to assess whether different ADs can be clustered using PRSs. Consistent clustering according to PRS composition may reflect the biological relevance of risk scores. The identification of a shared “autoimmune core” of polymorphisms could form the basis for general predictive systems for autoimmune disease risk. A binary matrix of PRS-associated genes was constructed to examine clustering patterns across different PRS sizes, and the functions of commonly shared genes were analyzed to evaluate their roles in AD pathogenesis. In addition, the function and role in the AD pathogenicity process was discussed for the most common shared genes.

## 2. Results

### 2.1. Most Frequently Encountered Genes

The analysis encompassed ten autoimmune diseases (ADs), including Multiple sclerosis, Celiac disease, Rheumatoid arthritis, Systemic Lupus Erythematosus, Psoriasis, T1DM, Asthma, Colitis, Crohn’s disease, and Thyroid disease (Hashimoto’s thyroiditis). Each disease was examined using polygenic scoring models (PRSs) with variants numbering less than 1000 extracted from the PGS Catalog database. Among the analyzed ADs, several genes were recurrently identified across different diseases. Shared genes with the highest frequency of occurrence were determined, indicating potential key players in the genetic architecture of autoimmune diseases (Table 2).

### 2.2. Review of SNPs in Shared Genes

We then annotated 453 SNPs in the top shared genes to find the most significant alterations that potentially have a significant impact on AD development. As expected, most of the SNPs found were intronic variants (Table S6).

Besides missense alterations, which have strong evidence of their impact in autoimmune disease pathogenesis in the literature and databases, there were a significant number of other alterations, including missense mutations in the introns and exons. However, information about the majority of them is limited. They have not been mentioned in the scope of autoimmune disease investigations, or annotated in public databases. Despite their usage in PRS, the level of evidence (LOE) in RegulomeDB (https://regulomedb.org/regulome-search/, assessed on 15 July 2025) is lower than 1e for a large number of them.

In Table 3, we provide the alteration and genes for the missense variants we found. Among them, there are several variants that were included in PRSs for certain autoimmune diseases, but the literature analysis showed a wider range of association for such variants, such as the rs30187 polymorphism of ERAP1: according to PRS, this alteration is common in psoriasis, but it is also associated with ankylosing spondylitis [56]. Association of one polymorphism with several autoimmune diseases is the primary purpose of the clustering analysis we performed and is described below.

Analysis of SNPs in the TSBP1 gene revealed that the majority of them are intronic variants. Intronic SNPs typically do not alter the amino acid sequence of the protein, but may affect RNA splicing and gene expression levels, which can influence functionality.

However, among all of the identified SNPs in the TSBP1 gene, only a few have the potential to alter the protein’s structure. Among them, only 4 SNPs—*rs3129941*, *rs560505*, *rs1265754*, and *rs9268384*—are Missense Variants, meaning that they cause a substitution of one amino acid for another in the protein sequence. Such changes may have functional consequences, as they could affect the structure or function of the TSBP1 protein. These SNPs may be associated with changes in phenotype or behavior related to the function of the TSBP1 gene, including its role in regulating immune processes.

### 2.3. Effect of PRS Size on Biological Impact

Polygenic risk scores (PRSs) are constructed from GWAS data and include variants statistically associated with disease, often with additional filtering, such as linkage considerations. The number of variants in different PRSs for the same disease can vary widely. It was hypothesized that PRSs containing fewer SNPs would consist of variants with stronger individual significance. Consequently, if autoimmune diseases share a common genetic core, PRSs with a small number of SNPs should more accurately capture biologically relevant genes strongly associated with immune system function. However, our analyses revealed that this prediction was not validated.

We analyzed the fraction of common genes based on PRS size in pairwise comparison for each AD pair (Tables S7–S9). Surprisingly, we found that the average fraction of common genes decreased from 4.75% in PRS sizes less than 1000 variants to 3.36% in PRS sizes less than 250 variants. We also assumed that the best performing PRS contained the most significant general autoimmune and disease-specific genes. In order to determine the ratio of common genes across various PRS scores, we formed a PRS group based on their performance. In this group, we included the top-5 PRSs for each disease based on their AUC score. For this group, the average fraction of common genes was 3.3% (Table S10). To visualize the distribution of ADs based on shared genes, we performed tSNE clustering based on the previously described binary matrix with each PRS size.

**Figure 1:**
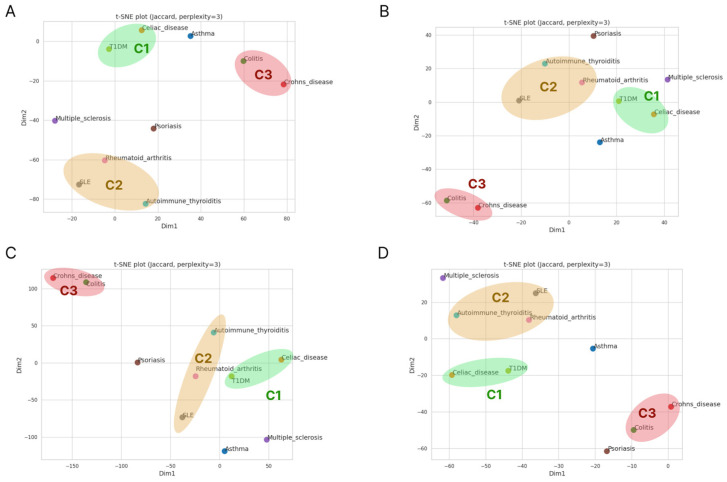
Clustering of autoimmune diseases based on common genes in polygenic risk scores. (**A**) For PRSs up to 1000 genes. (**B**) For PRSs up to 500 genes. (**C**) For PRSs up to 250 genes. (**D**) For PRSs with the best AUC per disease.

Initially, we identified three clusters of ADs: T1D was clustered with Celiac disease (C1); SLE was grouped with RA and Thyroid disease (C2), and Crohn’s disease and Colitis formed the third cluster (C3) (Figure 1A). Despite several changes in Euclidean distance between some diseases during PRS size decrease, ADs that formed clusters on PRS size 1000 grouped together on other PRS sizes (Figure 1B,C). T1D on PRS250 is located closer to RA than to Celiac disease, but, in general, the neighborhood remains stable. Interestingly, psoriasis could potentially be grouped with SLE, RA, and T1D, however, for the top AUC score PRS group, the distance between Psoriasis and C3 dramatically increased (Figure 1C).

It is important to note that tSNE clustering based on common PRS genes (Figure 1A) were grouped logically. T1D and Celiac disease, which compose C1, have a common genetic mechanism of pathogenesis [74]. Their correlation can also be defined by the greater co-occurrence that was observed in children with a T1D family history (HR: 2.80), *HLA-DR3/4* (HR: 1.94) and single-nucleotide polymorphism *rs3184504* at *SH2B3* (HR: 1.53).

C2 included RA and SLE, which are known as system immune disorders, affecting multiple systems and organs. These ADs were grouped with HT in our study, which has great differences in development and the organs that are affected. However, some studies have found that RA and HT share common features, such as alterations in CTLA-4, PTPN22, HLA-DRB, CD-40, and FOXP3 [75].

Crohn’s disease and ulcerative colitis (C3) are characterized by the enhanced recruitment and retention of effector macrophages, neutrophils, and T cells into the inflamed intestine, where they are activated and release proinflammatory cytokines [76]. The accumulation of effector cells in the inflamed intestine is a result of enhanced recruitment, as well as prolonged survival caused by decreased cellular apoptosis. Crohn’s disease is a predominantly T helper 1- and T helper 17-mediated process, while ulcerative colitis seems to be an atypical T helper 2 disorder. Despite this, Crohn’s disease and colitis clusterized together, regardless of the number of genes in the PRSs. Both of these diseases are inflammatory diseases of the bowel and they may have common mechanisms of pathology. This similarity can be explained by common genetic predisposers that were summarized in an article by Festen et al. [77].

It is interesting that the C1 cluster had the highest number of common genes across all PRS sizes (Table 4). We did not find any common genes across the three clusters that would reflect the absence of universal mechanisms of AD development. It is also important to note that the C2 cluster (RA, SLE, AIT) contained the smallest number of common genes. The number of shared genes in the PRS with the best GWAS performance in clusters 1 and 3 was comparable, unlike in cluster 2, where it was significantly lower. The full list of common genes in clusters and PRS sizes is presented in Supplementary Table S11.

To check whether cluster C2 had real similarity, we built heatmaps based on Jaccard metrics. This analysis showed that the C1 and C3 clusters were the most stable, despite the fact that in PRS size 1000, colitis had slightly better similarity with asthma or T1D. C2 showed an association between RA and SLE, but in PRS sizes 500 and 250, RA had significantly stronger similarity with T1D (as reflected in tSNE). It is worth noting that for SLE, the Jaccard metric showed the highest similarity with RA in all cases, despite RA having a higher similarity with T1D for PRS sizes below 1000. It is also of interest that AIT had good similarity with RA, less so with SLE, but on the top-5 AUC heatmap, AIT, RA, and SLE were clearly clustered together (Figures S1–S4). As such, we can conclude that PRS size has a partial impact on biological associations, primarily concerning generalized autoimmune diseases, with significantly less effect on tissue-specific diseases.

**Figure 2:**
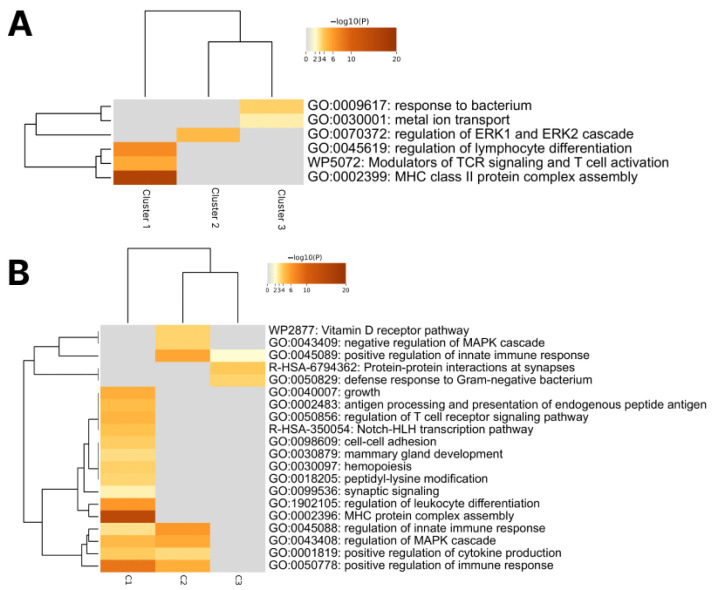
Pathway enrichment in identified clusters by common genes (**A**) across all PRS sizes (**B**) across PRS 1000.

As our next step, we tried to find differences in the pathological mechanisms between identified clusters. For this purpose, we used the Metascape tool (https://metascape.org/gp, assessed on 26 December 2025) to describe pathways enriched by genes identified as common. Firstly, we annotated common genes across all PRS sizes. This analysis showed that C1 (T1D and CD) was characterized by the enrichment of pathways associated with lymphocyte differentiation and activation (i.e., MHC assembly), C2 (SLE, RA, AIT) was associated with the regulation of the ERK1/2 cascade (plays an essential role downstream of immune receptors to elicit inflammatory gene expression in response to infection and cell or tissue damage), while C3 (Crohn’s disease and Colitis) had a response to bacterium and metal ion transport. Such heterogeneity between clusters may indicate several different mechanisms of immune system overactivation associated with ADs (Figure 2A).

To expand the dataset and test the hypothesis on a wider list of genes, we used the maximum PRS dimension (up to 1000). Pathway enrichment showed preservation and expansion of the effects we found on a smaller set of genes (Figure 2B). We found a link to Gram-negative bacteria response in C3, which are indeed described as triggers of colitis and Crohn’s disease [78]. In addition, there has been research pointing towards mechanisms of innate immune dysfunction in the development of Crohn’s disease [79]. In C2, a connection with MAPK pathway regulation has been described, with MAPK dysregulation being a common characteristic of these diseases [80,81,82]. C1 showed the highest number of enriched pathways involving antigen processing, MHC presentation, intracellular activation, and cytokine synthesis. Thus, we can say that the genes from the smallest set (250) successfully demonstrated the general direction of the pathogenic process, and expanding the set to 1000 provides some details about its mechanisms.

## 3. Discussion

The development of autoimmune diseases involves a complex interplay of genetic predisposition, environmental triggers, and dysregulation of the immune system. In this study, we explored the potential involvement of several genes in the pathogenesis of autoimmune disorders. Our findings shed light on how variations or alterations in these genes may contribute to the susceptibility and progression of autoimmune conditions.

We speculate that while the specific SNPs associated with these genes may differ depending on the specific disease, the genes themselves remain common across multiple autoimmune diseases. Indeed, significant SNPs usually change gene function [83]. Thus, the malfunction of the same gene caused by various SNPs may cause various ADs. We believe that this finding could support the hypothesis of a shared genetic basis for diverse autoimmune diseases. Potentially, this knowledge can be used for developing a common autoimmune risk score for the early prediction of immune system disbalance independent of tissue-specific genetic factors. To validate this hypothesis, we investigated several autoimmune diseases and conducted an analysis of genes and SNPs included in polygenic risk score (PRS) models for each disease.

We identified a list of common genes for the chosen ADs, which may be crucial for AD development. The most common gene is *TSBP1-AS1* (testis expressed basic protein 1 antisense RNA), which was associated with 8 out of 10 ADs. The functions of *TSBP1-AS1* have not been fully understood, but this long non-coding RNA (lncRNA) exhibits high expression levels in cells of the immune system [84]. According to the GWAS Catalog (https://www.ebi.ac.uk/gwas/, assessed on 15 June 2025), it is genetically associated with numerous immune-related and dermatological diseases. Furthermore, transcripts of *TSBP1-AS1* have the capability to interact with various molecules involved in gene regulation, including RNA-binding proteins (RBPs), messenger RNAs (mRNAs), and microRNAs (miRNAs) [85]. It is hypothesized that dysfunction of the *TSBP1-AS1* gene may lead to alterations in the regulation of immune responses. For example, if *TSBP1-AS1* expression is disrupted or if mutations in the gene alter its functionality, it could lead to improper activation or inhibition of immune cells, including T and B lymphocytes. Such changes could disrupt immune tolerance, which is a key aspect in the development of autoimmune diseases. Disruption of immune tolerance may lead to the activation of autoimmune cells directed against the body’s own tissues and organs. In addition, *TSBP1-AS1* is located near the HLA locus. lncRNAs in the MHC region are increasingly recognized as regulators of nearby immune genes [86]. As an antisense transcript, *TSBP1-AS1* may regulate TSBP1 expression via RNA interference, epigenetic modulation, or chromatin remodeling. These associations suggest that TSBP1-AS1 might act as an immune-related regulatory lncRNA, contributing to disease susceptibility by influencing immune-related gene expression.

Other previously mentioned genes also play a significant role in human immunology.

*HLA-DQB1*, *HLA-DRA*, *HLA-DPB1*, *TAP2*, *ERAP1*, *BTNL2*, and *HCG20* play an important role in the antigen presentation process; they are either part of the MHC complex or part of the mechanism that regulates peptide transport and processing before presentation [87].

The notable representation of genes located within the MHC region among the most frequently shared loci warrants careful consideration. This region is distinguished by remarkably elevated levels of linkage disequilibrium and a substantial accumulation of immune-related genes. Consequently, it is expected that this region will contribute disproportionately to shared genetic signals within the context of autoimmune polygenic risk scores. Consequently, the recurrent appearance of genes located in or near the MHC locus such as *TSBP1-AS1* and *TNXB* is likely driven, at least in part, by this regional genomic architecture.

It is important to note, however, that the shared signal is not restricted to MHC. Furthermore, an analysis of the data reveals the presence of several immune-relevant genes, including *IL2RA*, *SH2B3*, and *IRF5*, which are repeatedly observed across multiple scores. The presence of these non-MHC loci, in conjunction with the biologically coherent clustering of related autoimmune conditions, is consistent with a broader, genome-wide component to the shared genetic architecture. Taken together, these observations suggest that the identified common core reflects a combination of strong, regionally concentrated effects from MHC and a more distributed set of immunoregulatory pathways operating across the genome, rather than being solely attributable to linkage disequilibrium within a single genomic region.

*IRF5*, *SH2B3*, *GSDMB*, *CSMD1*, and *CLEC16A* are part of the autophagic regulation process and regulate inflammation [88]. *IL2RA* [89], *BACH2* [90], and *ZMIZ1* [91] support balance between immune activity and autoimmune processes. *RBFOX1* [92] plays a role in neuroimmune processes.

Some genes, such as *RBFOX1* [93] and *PTCHD1-AS* [94], are also associated with neural development and disorders. Upon initial observation, the identification of these genes, which are tentatively unrelated to the immune response, among genes associated with autoimmune diseases, appears to be paradoxical. However, there are several potential explanations for this phenomenon.

Firstly, pleiotropy is a well-established property of complex traits. Many loci contribute to multiple, seemingly unrelated, phenotypes by acting in fundamental cellular processes such as transcriptional regulation, RNA splicing, chromatin organization, or cell–cell communication. *RBFOX1*, although best known for its role in neuronal alternative splicing, regulates large splicing networks that are active across multiple tissues [95,96]. Perturbations in such global regulatory factors can influence immune cell differentiation, activation thresholds, or stress responses indirectly, thereby contributing to autoimmune susceptibility.

Secondly, neuroimmune and epithelial–immune crosstalk provides a plausible biological link. Autoimmune diseases frequently involve organs with dense neural innervation (gut, skin, and lung) and strong barrier functions. Genes implicated in neural development or synaptic organization may influence immune regulation via autonomic signaling, neuropeptide release, or modulation of epithelial integrity. In this context, loci initially discovered in neurodevelopmental disorders may reappear in autoimmune PRSs because they participate in shared signaling or regulatory pathways, rather than disease-specific immune mechanisms.

Thirdly, for non-coding loci such as *PTCHD1-AS*, the signal likely reflects regulatory effects, rather than gene-specific function. Long non-coding RNAs often act as cis-regulators of nearby genes or as components of broader chromatin domains. Variants mapped to *PTCHD1-AS* may tentatively tag regulatory regions that influence immune-relevant genes in a cell-type-specific manner t. This is particularly relevant in PRS construction, where intronic and intergenic SNPs with modest individual effects are aggregated.

*HCG20* and *LOC124901301* are lncRNA, the functions of which are mostly unknown, but they are localized in immune related parts of the DNA and may play a role in expression regulation [97].

Despite the active growth of GWAS studies, the comparison and application of developed risk scores remains a challenge. This is due to various factors, such as the heterogeneity of sequencing methods, development and testing on different populations, and different performance evaluation systems. In this article, we propose evaluating the preservation of biological meaning as a measure of the adequacy of the new PRS application, based on the genes included in it. To do this, we clustered autoimmune diseases based on the commonality of genes included in their risk scores. Our analysis showed that the biological relevance and uniform division of diseases into logical groups are preserved, regardless of the number of genes included in the risk score. It should be noted that similar mechanisms of pathogenesis and genetic predispositions have been described for the grouped diseases. This observation, which suggests a tendency to group autoimmune diseases into logical tissue- or pathogenetically specific clusters, was described in an article by Demela et al. [17]. However, when the PRS dimensionality was reduced, we found that the average percentage of common genes in a pairwise comparison of AD decreased. This may indicate that the broadest risk scores contain more genes that are directly related to the function of the immune system. Thus, for newly developed PRS, we propose evaluating their applicability and biological relevance through the analysis of the genes in their grouping, comparing them with other sets, namely those in the PGS catalog. Based on the size of the resulting PRS, we can estimate the average percentage of common autoimmune genes, assessing whether there are strong deviations towards more specific, or common immune-related genes. Additionally, the commonality of autoimmune genes can be evaluated. Their low number in PRSs should mean that, for some reason, GWAS did not identify the SNPs in these genes as significant, and it is worth rechecking the analysis.

In order to evaluate the functional relevance of the identified autoimmune disease clusters, an analysis was performed on the genes shared within each cluster across different PRS sizes, and a pathway enrichment analysis was conducted. Despite the minimal overlap of individual genes between clusters, genes consistently shared within a cluster formed functionally coherent groups enriched for immune-related processes. Distinct pathway signatures were observed across clusters. Of particular significance was the observation that these enrichment patterns remained consistent across PRS sizes, suggesting that incorporating additional variants primarily serves to reinforce existing biological pathways. The findings of this study indicate that the aggregation of autoimmune diseases is indicative of shared underlying biological mechanisms, which are captured by a limited set of core pathways. This observation contradicts the hypothesis that the aggregation is driven by PRS size or direct gene overlap between clusters.

Currently, there is an urgent need for the early diagnosis of autoimmune diseases in order to prevent them and calculate the therapeutic window for the use of pathogenetic therapy. The analysis of polymorphisms associated with ADs and their associated genes can serve as a useful tool for identifying the mechanisms of immune system function and its disorders, as well as a method for comparing the adequacy of the PRS being developed. In addition, the discovery of common genetic mechanisms for the development of different ADs or AD groups can serve as a basis for the development of new approaches for personalized pathogenetic therapy.

## 4. Materials and Methods

### 4.1. Identification of Autoimmune Diseases (ADs) and Data Collection

**Figure 3:**
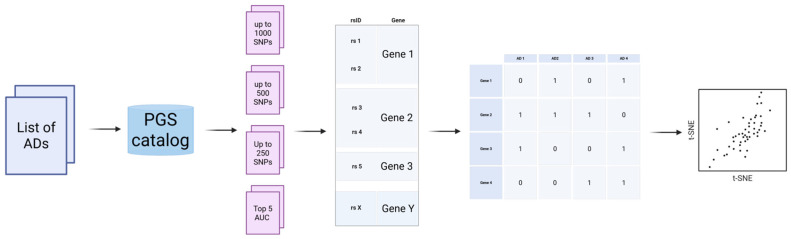
For each AD, SNPs from PRSs were obtained from the PGS Catalog. Different SNP sets were then created: all SNPs from PRSs containing up to 250 variants were combined into one set, then the same was carried out for PRSs with up to 500, and up to 1000 variants. Additionally, SNPs for the five top-performing PRSs based on their AUC were selected for each AD. Each SNP was mapped to its corresponding gene, and a binary matrix was constructed, indicating the presence of SNPs in specific genes for each disease. Finally, tSNE clustering was applied to each SNP set to identify common patterns among the autoimmune diseases.

Ten autoimmune diseases (ADs) were selected for investigation based on their prevalence and availability of data in public sources. For each disease, all SNPs included in polygenic scoring models (PRSs) listed in the PGS Catalog (https://www.pgscatalog.org/browse/scores/, assessed on 1 June 2025) were collected, stratified by models containing fewer than 1000 (PRS 1000), 500 (PRS 500), and 250 (PRS 250) variants (Figure 3). It should be noted that these data were nested: 1000 included 500, which in turn included 250. Limiting the size of the PRS to no more than 1000 variants was necessary to effectively manage the size of the datasets, limiting them to an acceptable number of genetic variants. To search the PGS Catalog, we utilized the name of the autoimmune disease as the key keyword.

Furthermore, we defined a group of the five best-performing PRSs for AD. Selection was based on the AUC metric available in the PGS Catalog, prioritizing scores validated within their original GWAS target population.

For each of the ten selected ADs, lists of single nucleotide polymorphisms (SNPs) included in the PGS were compiled. Subsequently, the SNP lists were merged by traits, and the corresponding genes for each rsID were identified using databases, such as dbSNP and NCBI Gene. From the PGS catalog, we obtained a total of 209 files each containing a list of SNPs of the certain chosen PRS. For the sets of PRSs with up to 1000 SNPs, a total of 58 files were available. Rheumatoid arthritis had the highest number of files (17). Overall, there were 9911 unique SNPs across all files. To work with the table, we utilized the Python programming language (v. 3.0) along with the pandas library.

### 4.2. Preparation of Gene-SNP Correspondence Table for Each AD

Then, a table was compiled, indicating the SNPs (rsID) from all chosen PRSs and their weights for each position as well as the corresponding gene and disease for each chosen PRS. The corresponding gene was identified through automated annotation using the NCBI dbSNP database. Specifically, a custom Python workflow was implemented to ensure reproducibility and consistency across all PRSs. Summary statistics files in .txt format were parsed to extract the rsID column. For each variant, the script queried dbSNP via the Entrez Direct utilities (esearch, esummary, and xtract) to retrieve associated gene symbols (field: GENE_E/NAME). Gene names were deduplicated and appended to the corresponding rsID entry, and the resulting tables were consolidated into a final reference file (Table S1).

### 4.3. Analysis of Intersections and Common Genes

To assess the genetic similarities among different autoimmune diseases, a binary matrix (Gene-AD) was constructed for summarized data for each of the 3 PRS sizes (1000, 500, and 250). Presence of the variant in a gene for a certain AD was defined as 1, absence was defined as 0 (Table S2). Then, for each PRS size group and for top-performing PRSs, we calculated the number and fraction of common and individual genes for each pair in the chosen AD list (Tables S3–S5).

### 4.4. Identification of Genes in Which SNPs Are Associated with Multiple AD Pathogenesis

To identify the genes in which SNPs are associated with multiple AD pathogenesis, frequencies of gene and SNP occurrence in the dataset were calculated. The topmost frequently encountered genes were selected for further analysis. First, all missense variants were analyzed for pathogenicity using ClinVar annotations [98] and Regulome DB (https://regulomedb.org) [99]. Then, all others variants were analyzed: variants with regulome scores higher than 1f (eQTL/caQTL + TF binding/chromatin accessibility peak, per Regulome DB), which is the most common score for all analyzed variants, were taken to further analysis, including a literature check, to determine whether these variants could be associated with immune diseases, and how they can affect protein function (Table S6). Most SNPs are not annotated in ClinVar and have no pathogenicity status, so previous research was the most efficient way to find reliable SNPs in PRS.

### 4.5. Data Processing and Clustering

For data analysis, we used Python version 3.0 and the following packages: pandas, numpy, scikit-learn, matplotlib, and seaborn. Dimensionality reduction and visualization of disease similarity were performed using t-distributed stochastic neighbor embedding (t-SNE). The analysis was based on a binary matrix representing the presence or absence of genes across immune-mediated diseases. t-SNE was applied to the transposed matrix, treating diseases as data points and genes as binary features.

We used Jaccard distance as the similarity metric and set perplexity to 3 to account for the small number of samples (diseases). The resulting two-dimensional embeddings were visualized using the Python v.3.0 (the Seaborn and Matplotlib libraries), with each disease labeled and color-coded to highlight potential clustering patterns.

## 5. Conclusions

The current paradigm of genetic predisposition to autoimmune diseases is gradually shifting from a primary focus on polymorphisms within immune genes and their proximal regulatory regions toward the identification of intergenic variants with potentially strong effects [100,101]. Our study shows that the saturation of PRS with a large number of detected signals does not affect the biological significance and has a limited impact on the performance of the risk scale. From this perspective, a more promising strategy may be to prioritize the identification of the most informative variants that are either common to the development of autoimmune mechanisms, or specific to a particular autoimmune disease.

However, identifying the shared genetic features underlying ADs that cause this particular clustering may enable the development of a generalized framework for estimating the long-term risk of developing ADs. Such an approach could further contribute to predicting which clinical manifestations are more likely to arise in a given individual by linking genetic profiles to specific autoimmune clusters, thereby providing a biologically grounded basis for stratification beyond disease-specific risk scores.

While the findings suggest that biological interpretability remains consistent across PRS sizes, this consistency does not equate to universal equivalence in application. The results of the present study emphasize a critical, context-dependent distinction between the extraction of shared biological meaning and the achievement of maximal predictive utility. Compact, targeted PRSs, enriched for core pathways, are likely sufficient for mechanistic insight and population stratification. Conversely, larger PRSs, which aggregate a more comprehensive spectrum of polygenic variance, are still expected to be superior for individual-level risk prediction where accuracy is critical. Consequently, the optimal PRS design should be guided by its primary objective.

## Figures and Tables

**Table 1:** Incidence and Prevalence of Polygenic Autoimmune Diseases.

Disease	Target	Incidence	Prevalence
T1DM	Pancreas	15 per 100,000 people [24]	0.12% in Europe [24]
Crohn’s disease	Gastrointestinal tract	3–20 per 100,000 [25]	0.2% to 0.3% [26]
Ulcerative colitis	Gastrointestinal tract	20 per 100,000 [27]	0.3% [27]
Systemic lupus erythematosus	Multiple organs	1–8.7 per 100,000 [28]	0.03% to 0.05% [28]
Hashimoto’s thyroiditis	Thyroid gland	80 (male)–350 (female) cases per 100,000 [29]	7.5% [30]
Psoriasis	Skin	78.9 per 100,000 [31] 30.3–321.0 per 100,000 person years [32]	0.14–1.99% [32]
Rheumatoid arthritis	Synovial tissue	208.8 cases (186.8–241.1) per 100,000 [33]	2.45% [33]
Asthma	Lungs	477.92 per 100,000 [34]	10.0–13.2% [35]
Celiac disease	Small intestine	7.8 (male)–17.4 (female) per 100,000 person years [36]	1.4% [37]
Multiple sclerosis	Central nervous system	2.1 [95% CI: 2.09, 2.12] per 100,000 person years [38]	2.8 million (0.04%) [38]

**Table 2:** Most common genes in PRS contain up to 1000 variants across ADs.

Gene	Diseases	Comment
*TSBP1-AS1*	Asthma, Celiac_disease, Colitis, Multiple_sclerosis, Psoriasis, Rheumatoid_arthritis, T1DM, Thyroid_disease	*TSBP1-AS1* is a long non-coding RNA (lncRNA) that is thought to regulate the expression of proximal genes, including immune-related genes within the MHC region. Its exact function is still being explored, but it may play a role in autoimmune disease susceptibility (https://www.ncbi.nlm.nih.gov/gene/10665, assessed on 15 July 2025).
*GSDMB*	Asthma, Colitis, Crohn’s_disease, Psoriasis, Rheumatoid_arthritis, T1DM, Thyroid_disease	*GSDMB* (Gasdermin B) is involved in cell death processes like pyroptosis, a form of programmed cell death linked to inflammation. It has been associated with asthma, inflammatory bowel disease, and some cancers [39].
*TNXB*	Celiac_disease, Multiple_sclerosis, Psoriasis, Rheumatoid_arthritis, SLE, T1DM, Thyroid_disease	The *TNXB* gene encodes tenascin-XB, a member of the tenascin family of extracellular matrix glycoproteins. Mutations are linked to Ehlers–Danlos syndrome and may affect immune regulation [40].
*IL2RA*	Asthma, Colitis, Multiple_sclerosis, Psoriasis, Rheumatoid_arthritis, SLE, T1DM	*IL2RA* encodes the alpha chain of the interleukin-2 receptor, crucial for T cell function, and immune regulation. Variants in IL2RA are linked to autoimmune diseases like type 1 diabetes and multiple sclerosis [41].
*SH2B3*	Asthma, Celiac_disease, Psoriasis, Rheumatoid_arthritis, SLE, T1DM, Thyroid_disease	The *SH2B3* gene, also known as LNK, encodes a member of the SH2B adaptor protein family, which plays a pivotal role in hematopoiesis and acts as a key negative regulator of cytokine signaling. The protein product of *SH2B3* is involved in various signaling pathways initiated by growth factors and cytokines, and its function is critical for the proper regulation of these pathways (https://www.ncbi.nlm.nih.gov/gene/10019, assessed on 15 July 2025).
*IRF5*	Asthma, Colitis, Crohn_disease, Psoriasis, Rheumatoid_arthritis, SLE, Thyroid_disease	*IRF5* (Interferon Regulatory Factor 5) is a transcription factor involved in the regulation of type I interferon and pro-inflammatory cytokines. It plays a central role in immune response and has been implicated in lupus and other autoimmune conditions [42].
*TSBP1*	Asthma, Celiac_disease, Multiple_sclerosis, Psoriasis, Rheumatoid_arthritis, T1DM, Thyroid_disease	*TSBP1* is a gene located within the major histocompatibility complex (MHC) region, and, while its precise biological function is not fully understood, it is thought to be involved in transcriptional regulation or chromatin organization. It may also have a regulatory role in immune-related pathways due to its proximity to immune genes. Research into its function is ongoing, particularly regarding its potential link to autoimmune diseases [43].
*TAP2*	Asthma, Celiac_disease, Colitis, Psoriasis, Rheumatoid_arthritis, T1DM, Thyroid_disease	*TAP2* encodes a membrane-associated protein that plays a crucial role in the immune system by forming a heterodimer with ABCB2, another transporter protein, to facilitate the transport of peptides from the cytoplasm into the endoplasmic reticulum (https://www.ncbi.nlm.nih.gov/gene/6891, assessed on 15 July 2025) [44].
*BACH2*	Asthma, Multiple_sclerosis, Rheumatoid_arthritis, SLE, T1DM, Thyroid_disease	The *BACH2* gene is a critical regulator of the primary adaptive immune response, with a role in the development and function of T cells and B cells (https://www.ncbi.nlm.nih.gov/gene/60468, assessed on 15 July 2025). In the immune system, BACH2 has been linked to the regulation of gene expression through epigenetic mechanisms, such as the methylation of histone H3 at lysine 79 (H3K79me), mediated by DOT1L [45].
*HLA-DQB1*	Asthma, Celiac_disease, Colitis, Rheumatoid_arthritis, T1DM, Thyroid_disease	*HLA-DQB1* is a key gene in the MHC class II region that helps present antigens to CD4+ T cells. Its alleles are strongly associated with autoimmune diseases, such as celiac disease and type 1 diabetes [46].
*HLA-DRA*	Asthma, Celiac_disease, Multiple_sclerosis, Psoriasis, Rheumatoid_arthritis, T1DM	*HLA-DRA* encodes the alpha chain of the *HLA-DR* antigen, which is a major histocompatibility complex (MHC) class II molecule (https://www.ncbi.nlm.nih.gov/gene/3122, assessed on 15 July 2025).
*RBFOX1*	Asthma, Celiac_disease, Colitis, Crohn_disease, Psoriasis, T1DM	*RBFOX1* is an RNA-binding protein that regulates alternative splicing in the nervous system and heart. It has been linked to neurodevelopmental disorders such as autism, epilepsy, and schizophrenia [47].
*PTCHD1-AS*	Asthma, Celiac_disease, Colitis, Psoriasis, Rheumatoid_arthritis, T1DM	*PTCHD1-AS* is a non-coding RNA that may regulate the *PTCHD1* gene, which is involved in neural development. Variants in this region are associated with intellectual disability and autism spectrum disorders [48].
*LOC124901301*	Asthma, Celiac_disease, Multiple_sclerosis, SLE, T1DM, Thyroid_disease	*LOC124901301* is a predicted or uncharacterized genomic locus; its biological function is currently unknown. Further research is needed to determine its role.
*HLA-DPB1*	Asthma, Celiac_disease, Multiple_sclerosis, Rheumatoid_arthritis, T1DM, Thyroid_disease	*HLA-DPB1* is part of the MHC class II complex involved in presenting peptides to T-helper cells. It has associations with various immune responses, including transplant compatibility and autoimmune diseases.
*CSMD1*	Celiac_disease, Colitis, Psoriasis, Rheumatoid_arthritis, T1DM, Thyroid_disease	The gene product of *CSMD1* functions as a complement control protein. *CSMD1* is thought to act as a tumor suppressor and is involved in complement system regulation and neural development. It has been implicated in schizophrenia and some cancers [49].
*HCG20*	Celiac_disease, Multiple_sclerosis, Psoriasis, Rheumatoid_arthritis, T1DM, Thyroid_disease	*HCG20* is a non-coding RNA gene located in the MHC region on chromosome 6; its function is not well-understood. It may be involved in regulating immune gene expression or linked to disease susceptibility through genetic proximity (https://www.ncbi.nlm.nih.gov/gene/?term=HCG20, assessed on 15 July 2025).
*BTNL2*	Celiac_disease, Colitis, Multiple_sclerosis, Psoriasis, Rheumatoid_arthritis, T1DM	*BTNL2* encodes a butyrophilin-like protein that modulates T-cell activation and immune response. Variants are associated with sarcoidosis and other inflammatory conditions [50].
*ERAP1*	Asthma, Crohn’s_disease, Psoriasis, Rheumatoid_arthritis, SLE, Thyroid_disease	*ERAP1* (Endoplasmic Reticulum Aminopeptidase 1) trims peptides for presentation on MHC class I molecules. It is strongly associated with autoimmune diseases, like ankylosing spondylitis [51,52].
*CNTN5*	Asthma, Crohn’s_disease, Multiple_sclerosis, Rheumatoid_arthritis, T1DM, Thyroid_disease	*CNTN5* encodes Contactin-5, a neural adhesion molecule important for brain development. It has been linked to autism spectrum disorders and cognitive dysfunction [53].
*ZMIZ1*	Celiac_disease, Multiple_sclerosis, Psoriasis, Rheumatoid_arthritis, SLE, T1DM	*ZMIZ1* (zinc finger, MIZ-type containing 1) is a gene that encodes a protein belonging to the PIAS (protein inhibitor of activated STAT) family, which plays a crucial role in regulating the activity of various transcription factors, including the androgen receptor, Smad3/4, and p53. The protein is also implicated in the process of sumoylation. The gene also has a connection to the immune system, as evidenced by its relationship with the novel soluble immune system factor ISRAA. ISRAA is nested within intron 6 of the mouse Zmiz1 gene and has been shown to play a role in modulating anti-infection immunity by downregulating T-cell activation [54].
*CLEC16A*	Asthma, Multiple_sclerosis, Rheumatoid_arthritis, SLE, T1DM, Thyroid_disease	*CLEC16A* encodes a protein involved in autophagy and antigen presentation processes. It is a strong susceptibility gene for autoimmune diseases including type 1 diabetes and multiple sclerosis [55].

**Table 3:** Most significant SNPs across the top shared genes according to the literature analysis and their effect on molecular pathways, as shown in the Regulome database.

rsID	Gene	Source	Type	Location	LOE Regulome DB	Description	Source
*rs41521946*	*BTNL2*	PGS001306	missense	intron	1f	*rs41521946* was shown to correlate with the development of knee osteoarthritis.	[57]
*rs2076530*	*BTNL2*	PGS001309 PGS001310	missense	intron	1f	A study has shown that, in addition to being associated with sarcoidosis, *rs2076530* may play a role in rheumatoid arthritis.	[58]
						A transition constituting *rs2076530* leads to the use of a cryptic splice site located 4 bp upstream of the affected wild-type donor site. Transcripts of the risk-associated allele have a premature stop in the spliced mRNA. The resulting protein lacks the C-terminal IgC domain and transmembrane helix, thereby disrupting the membrane localization of the protein, as shown in experiments using green fluorescent protein and V5 fusion proteins.	[59]
*rs27044*	*ERAP1*	PGS002293	missense	exon	1f	According to meta analysis, the *rs27044* polymorphism was significantly associated with ankylosing spondylitis susceptibility in the overall population: *rs27044*, G versus C, OR = 1.24, 95% CI 1.16–1.33, *p* < 0.001. When stratified by ethnicity, *rs27044* appeared to be significantly correlated with AS in both Asians and Caucasians.	[56]
						This polymorphism is also known to be associated with psoriasis.	[60]
*rs26653*	*ERAP1*	PGS001345	missense	exon	1f	This polymorphism is known to be associated with psoriasis.	[60]
*rs2549782*	*ERAP1*	PGS001043	missense	intron	1f	This polymorphism is known to be associated with ankylosing spondylitis.	[61]
*rs30187*	*ERAP1*	PGS001312	missense	exon	1b	Significant epistatic interaction was observed between HLA-C*06 and the SNP (*rs27044*) located at the peptide-binding cavity of ERAP1. Evolutionary conservation analysis among mammals showed confinement of Lys528 and Gln730 within highly conserved regions of ERAP1 and suggested the possible detrimental effect of this allele in ERAP1 regulation.	[62]
						It was shown that there is a significant association between *rs30187* polymorphisms and psoriasis susceptibility (T vs. C, OR = 1.23, 95% CI: 1.15–1.32, *p* < 0.00001).	[63]
*rs2305480*	*GSDMB*	PGS000037	missense	exon	1f	GWAS risk loci study showed that this polymorphism is a risk factor for asthma development.	[64]
*rs2305479*	*GSDMB*	PGS004252	missense	exon	1f	It was shown that this polymorphism is associated with allergic rhinitis in the Chinese population.	[65]
*rs9277471*	*HLA-DPB1*	PGS001301	missense	intron	1b	It was shown that that *rs9277471* is associated with multiple sclerosis in GWAS pathway analysis.	[66]
*rs1130399*	*HLA-DQB1*	PGS001306	missense	intron	1f	It was shown that that *rs1130399* has an association with Alzheimer’s disease.	[67]
*rs1130368*	*HLA-DQB1*	PGS001310	missense	intron	1f	It was shown that this polymorphism is associated with inflammatory bowel in the Japanese population.	[68]
*rs7192*	*HLA-DRA*	PGS001313	missense	intron	1f	A psoriasis genome-wide association study (GWAS) dataset that included 436,192 SNPs in 1409 psoriasis cases and 1436 controls of European descent, and a BD GWAS dataset that contained 310,324 SNPs in 1215 BD cases; 1278 controls were used in this study. Identify candidate causal SNPs and pathways (ICSNPathway) analysis was applied to the GWAS datasets, which identified 15 candidate causal SNPs and 28 candidate causal pathways. The top five candidate causal SNPs were *rs1063478* (*p* = 1.45 × 10^−10^), *rs8084* (*p* = 2.20 × 10^−8^), *rs7192* (*p* = 5.18 × 10^−8^), *rs20541* (*p* = 5.30 × 10^−6^), and *rs1130838* (*p* = 5.65 × 10^−6^), which with the exception of *rs20541* [interleukin (IL)-13] are at the human leukocyte antigen (HLA) loci.	[69]
						Since this SNP (*rs7192*, HLA00662.1:g.4276G>T p.Val217Leu) lies within exon 4, in the region encoding the cytoplasmic tail, the resulting protein is effectively monomorphic. For this reason, in-depth studies on *HLA-DRA* and its function have been limited. However, analysis of sequences from the 1000 Genomes Project and preliminary data from our lab revealed an unrepresented polymorphism within *HLA-DRA*, suggesting a more complex role within the MHC than previously assumed. This study focused on elucidating the extent of *HLA-DRA* polymorphism, and extending our understanding of the gene’s role in *HLA-DR*~*HLA-DQ* haplotypes. Ninety-eight samples were sequenced for full-length *HLA-DRA*, and from this analysis, we identified 20 novel SNP positions in the intronic sequences within the 5711 bp region represented in IPD-IMGT/HLA. This polymorphism gives rise to at least 22 novel *HLA-DRA* alleles, and the patterns of intronic and 3’ UTR polymorphism correspond to *HLA-DRA*~*HLA-DRB345*~*HLA-DRB1*~*HLA-DQB1* haplotypes. The current understanding of the organization of the genes within the *HLA-DR* region assumes a single lineage for the *HLA-DRA* gene, as opposed to multiple gene lineages, such as in *HLA-DRB*.	[70]
*rs3184504*	*SH2B3*	PGS001345	missense	intron	1f	It was shown that *rs3184504* has an association with celiac disease.	[71]
						The rs3184504*T allele is associated with a loss-of-function amino acid change (p.R262W) in the adaptor protein *SH2B3*, a likely causal variant. The peritoneal infiltrating cells exhibited augmented phagocytosis in *Sh2b3* −/− mice with enriched recruitment of Ly6Chi inflammatory monocytes despite equivalent or reduced chemokine expression. Rapid cycling of monocytes and progenitors occurred uniquely in Sh2b3 −/− mice following CLP, suggesting augmented myelopoiesis.	[72]
*rs185819*	*TNXB*	PGS001296	missense	intron	1f	GWAS association with systemic lupus erythematosus and rheumatoid arthritis.	[73]

**Table 4:** Number of common genes across clusters in different analysis sets.

Cluster	PRS 250 Genes	PRS 500 Genes	PRS 1000 Genes	Common in Across PRS Sizes	Common in Top-5 AUC
C1—Celiac Disease, T1D	21	128	94	18	37
C2—RA, SLE, AIT	9	12	23	8	6
C3—Colitis, Crohn’s disease	16	18	40	16	40
Common gene between clusters	0	0	0	0	0

## Data Availability

The data presented in this study are available in the PGS Catalog at https://www.pgscatalog.org. These data were derived from the following resources available in the public domain: https://www.pgscatalog.org.

## Supplementary Materials

The following supporting information can be downloaded at: https://www.mdpi.com/article/10.3390/ijms27010543/s1.
